# Deep Learning for Detecting Brain Metastases on MRI: A Systematic Review and Meta-Analysis

**DOI:** 10.3390/cancers15020334

**Published:** 2023-01-04

**Authors:** Burak B. Ozkara, Melissa M. Chen, Christian Federau, Mert Karabacak, Tina M. Briere, Jing Li, Max Wintermark

**Affiliations:** 1 Department of Neuroradiology, MD Anderson Cancer Center, 1400 Pressler Street, Houston, TX 77030, USA; 2 Faculty of Medicine, University of Zurich, Pestalozzistrasse 3, CH-8032 Zurich, Switzerland; 3 Department of Neurosurgery, Mount Sinai Health System, 1468 Madison Avenue, New York, NY 10029, USA; 4 Department of Radiation Physics, MD Anderson Cancer Center, 1515 Holcombe Boulevard, Houston, TX 77030, USA; 5 Department of Radiation Oncology, MD Anderson Cancer Center, 1515 Holcombe Boulevard, Houston, TX 77030, USA

**Keywords:** artificial intelligence, deep learning, brain metastasis, magnetic resonance imaging, pooled analysis

## Abstract

**Simple Summary:**

Manual detection and delineation of brain metastases are time consuming and variable. Studies have therefore been conducted to automate this process using imaging studies and artificial intelligence. To the best of our knowledge, no study has conducted a systematic review and meta-analysis on brain metastasis detection using only deep learning and MRI. As a result, a systematic review of this topic is required, as well as an assessment of the quality of the studies and a meta-analysis to determine the strength of the current evidence. The purpose of this study was to perform a systematic review and meta-analysis of the performance of deep learning models that use MRI to detect brain metastases in cancer patients.

**Abstract:**

Since manual detection of brain metastases (BMs) is time consuming, studies have been conducted to automate this process using deep learning. The purpose of this study was to conduct a systematic review and meta-analysis of the performance of deep learning models that use magnetic resonance imaging (MRI) to detect BMs in cancer patients. A systematic search of MEDLINE, EMBASE, and Web of Science was conducted until 30 September 2022. Inclusion criteria were: patients with BMs; deep learning using MRI images was applied to detect the BMs; sufficient data were present in terms of detective performance; original research articles. Exclusion criteria were: reviews, letters, guidelines, editorials, or errata; case reports or series with less than 20 patients; studies with overlapping cohorts; insufficient data in terms of detective performance; machine learning was used to detect BMs; articles not written in English. Quality Assessment of Diagnostic Accuracy Studies-2 and Checklist for Artificial Intelligence in Medical Imaging was used to assess the quality. Finally, 24 eligible studies were identified for the quantitative analysis. The pooled proportion of patient-wise and lesion-wise detectability was 89%. Articles should adhere to the checklists more strictly. Deep learning algorithms effectively detect BMs. Pooled analysis of false positive rates could not be estimated due to reporting differences.

## 1. Introduction

Brain metastases (BMs) are observed in nearly 20% of adult cancer patients [1]. They are the most common type of intracranial neoplasm in adults [2]. Although the brain parenchyma is the most common intracranial metastatic site, BMs frequently occur in conjunction with metastases to other sites such as the cranium, dura, or leptomeninges [3]. Therefore, making accurate diagnosis of BM is critical.

Contrast-enhanced magnetic resonance imaging (MRI) is the preferred imaging examination for diagnosing BMs. It is more sensitive than either nonenhanced MRI or computed tomography (CT) in detecting lesions [4,5,6,7]. Although whole-brain radiotherapy (WBRT), which can cause cognitive decline, has historically been the mainstay of radiotherapy for the treatment of BMs, stereotactic radiosurgery (SRS) has now become the standard of care in many clinical situations. In 2014, a multi-institutional prospective study demonstrated that SRS without WBRT in patients with five to ten brain metastases is non-inferior to that in patients with two to four brain metastases [8]. Therefore, SRS plays a more prominent role today and new treatment methods, such as hippocampal avoidance-WBRT and newer systemic agents, are being developed [9]. Before SRS, in the current standard practice, each BM must be correctly detected and manually delineated for treatment planning, which is time consuming and subject to considerable inter- and intra-observer variability [10].

Because manual detection and delineation of BMs is time consuming and variable, research has been done to make this process automated using imaging studies with the help of artificial intelligence [11]. Deep learning, a type of representation learning method, has emerged as the cutting-edge machine learning method [12]. Because it does not require an intermediary feature extraction or an engineering phase to learn the relationship between the input and the output, deep learning has accelerated the development of computer-aided detection in various fields [13]. Furthermore, with advances in network architectures and increased imaging data quantity and quality in recent years, the performance of deep learning-based approaches has been greatly enhanced [14]. A meta-analysis of 12 studies examined computer-aided detection in BMs and showed a pooled proportion of detectability of 0.90 (95% confidence interval [CI], 85–93%) [11]. However, they included articles that used machine learning or deep learning algorithms, totaling twelve studies in their meta-analysis, with only seven implementing deep learning.

To the best of our knowledge, no study in the literature has performed a systematic review and meta-analysis on BM detection with exclusively deep learning using MRI. As a result, it is necessary to conduct a systematic review of this topic, assess the quality of the studies, and run a meta-analysis to evaluate the strength of the current evidence. This study aimed to conduct a systematic review and meta-analysis of the performance of deep learning models that implement MRI in detecting BMs in cancer patients.

## 2. Materials and Methods

This study followed the Preferred Reporting Items for Systematic Reviews and Meta-Analyses extension for Diagnostic Test Accuracy Studies (PRISMA-DTA) [15]. Checklists for PRISMA-DTA for Abstracts and PRISMA-DTA are available online in Supplemental Table S1 and Supplemental Table S2, respectively. The study has not been registered. This study is exempt from ethical approval of the Institutional Review Board since the analysis only involves anonymized data, and all the included studies have received local ethics approval.

### 2.1. Literature Search

A systematic search of articles was performed by two independent readers (B.B.O. and M.K., with more than 2 years and 4 years of experience in conducting systematic reviews and meta-analyses, respectively) from inception until September 30, 2022. The following search terms were used to search MEDLINE: (“deep learning” OR “deep architecture” OR “artificial neural network” OR “convolutional neural network” OR “convolutional network” OR CNN OR “recurrent neural network” OR RNN OR Auto-Encoder OR Autoencoder OR “Deep belief network” OR DBN OR “Restricted Boltzmann machine” RBM OR “Long Short Term Memory” OR “Long Short-Term Memory” OR LSTM OR “Gated Recurrent Units” OR GRU) AND ((“brain metastasis”) OR (“brain metastases”) OR (“metastatic brain tumor”) OR (“intra-axial metastatic tumor”) OR (“cerebral metastasis”) OR (“cerebral metastases”)). The search strategy was then adapted for EMBASE and Web of Science before searching these databases.

### 2.2. Study Selection

All search results were exported to the Rayyan online platform [16]. After removing duplicates, two authors (B.B.O. and M.K.) independently screened titles and abstracts using the Rayyan platform and reviewed the full text of potentially relevant articles. A senior author compared and examined the results of each search and analysis step (M.W., 25 years of experience in neuroradiology). Any disagreement was resolved via discussion with the senior author (M.W.).

Articles were included based on the satisfaction of all the following criteria: (I) inclusion of patients with BMs; (II) deep learning using MRI images was applied to detect the BMs; (III) sufficient data were present in terms of detective performance of the deep learning algorithms; (IV) original research articles.

Articles were excluded if they fulfilled any of the following criteria: (I) reviews, letters, guidelines, editorials, or errata; (II) case reports or series with less than 20 patients; (III) studies with overlapping cohorts; (IV) insufficient data in terms of detective performance of the deep learning algorithms; (V) machine learning, not deep learning, was used to detect BMs; (VI) articles not written in English. In the case of overlapping cohorts, the article with the largest sample size was included. If articles have the same number of patients, the most recent one was preferred.

### 2.3. Data Extraction

The following variables were collected by the two authors (B.B.O. and M.K.): (I) study characteristics (first author, publication year, study design, number of patients in each dataset, sex of patients in each dataset, number of metastatic lesions in each dataset, mean or median size of lesions, reference standard for metastasis detection, validation method, and primary tumors); (II) deep learning details and statistics (detectability, false positive rate, deep learning algorithm, and data augmentation); (III) MRI and scanner characteristics; and (IV) inclusion and exclusion criteria of each study.

### 2.4. Quality Assessment

Two authors (B.B.O. and M.K.) independently performed the quality assessments using the Checklist for Artificial Intelligence in Medical Imaging (CLAIM) and Quality Assessment of Diagnostic Accuracy Studies-2 (QUADAS-2) [17,18]. The CLAIM is a new 42-item checklist for evaluating artificial intelligence studies in medical imaging. For each item, studies were given a score of 0 or 1 on a 2-point scale. The CLAIM score was calculated by adding the scores from each study. The items were all equally weighted. QUADAS-2 was used to assess four domains: (I) patient selection, (II) index test, (III) reference standard, and (IV) flow and timing. During the quality assessment, any disagreements were resolved with the assistance of the senior author.

### 2.5. Meta-Analysis

The primary goal was to assess the detectability (sensitivity) of deep learning algorithms in detecting BMs. For the pooled proportion analysis of detectability of all included studies in the meta-analysis, the reported sensitivity per study was multiplied by the total number of patients in each study to estimate the number of detected events. In other words, reported sensitivity was converted to patient-wise sensitivity. Another pooled proportion analysis of detectability was performed for studies reporting their sensitivity lesion-wise. The detectability of each study in this group was calculated by dividing the number of correctly identified metastases in the test set (using given true positive values or calculated with reported sensitivities) by the total number of metastases in the test set. Following statistical analyses were performed in both pooled analyses.

**Figure 1:**
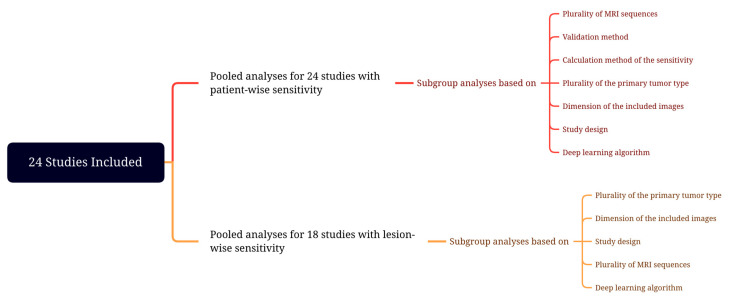
Meta-analysis design.

The pooled proportion analysis of detectability estimates with 95% CIs was performed with the random-effects model. The random-effects model, using the inverse variance method, was chosen since it captures uncertainty due to heterogeneity among studies [19,20]. The Freeman–Tukey double arcsine transformation was performed to stabilize the variances before pooling. Forest plots were created to provide a visual representation of the results. Meta-regression analyses were performed to determine if training size was associated with the sensitivity. Heterogeneity among all included studies was evaluated using Q-test with *p* < 0.05, suggesting the presence of study heterogeneity and I2 statistics. I2 values were defined as follows: heterogeneity that might not be important (0–25%), low heterogeneity (26–50%), moderate heterogeneity (51–75%), and high heterogeneity (76–100%) [21]. Since heterogeneity might indicate subgroup effects, we also explored heterogeneity in the pooled results using subgroup analysis [22]. Subgroup analyses were conducted in terms of the validation method used in the study (split training-test sets versus cross-validation), the plurality of MRI sequences utilized (single sequence versus multi-sequence), the calculation method of the reported sensitivity rates (lesion-wise versus other [patient-wise, voxel-wise]), the plurality of the primary tumor type (single versus multiple), dimension of the included images (2D versus 3D versus both), study design (single-center versus multi-center), and deep learning algorithms (DeepMedic versus U-Net versus others)—Figure 1. Meta-regression analyses were also performed to determine if training size was associated with heterogeneity. Publication bias occurs when the findings of a study influence the study’s likelihood of publication. The Egger method was applied to test the funnel plot asymmetry for publication bias [23]. R version 4.2.1 was used for all statistical analysis, and the function metaprop from package meta was utilized to perform meta-analysis and generate pooled estimates [24]. An alpha level of 0.05 was considered statistically significant.

## 3. Results

### 3.1. Literature Search

**Figure 2:**
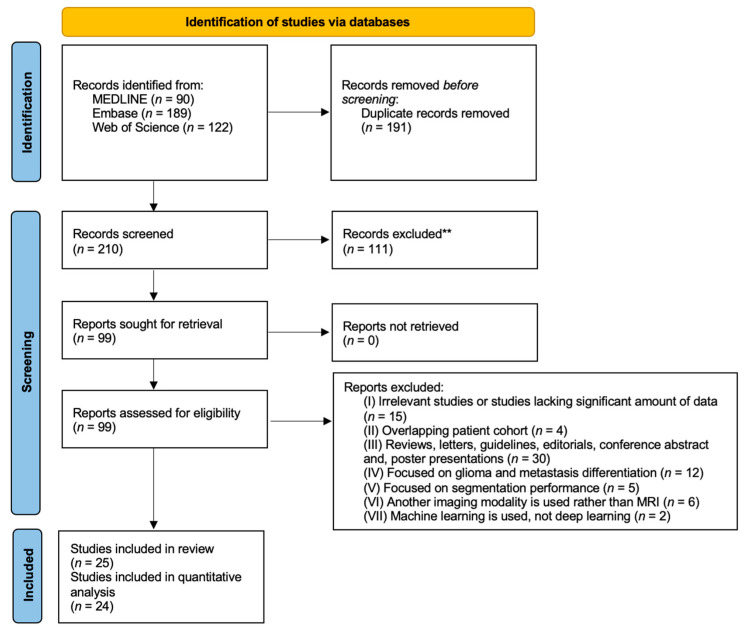
Study selection process.

The study selection process is shown in the PRISMA flowchart (Figure 2). The literature search yielded 401 studies: 90 from MEDLINE, 189 from EMBASE, and 122 from Web of Science. After removing 191 duplicate articles, the remaining 210 were screened based on their title and abstract on the Rayyan platform, and 111 were excluded. Full texts of the remaining 99 articles were acquired and reviewed.

Seventy-four articles were excluded because: irrelevant studies or studies lacking significant amount of data (*n* = 15); overlapping patient cohort (*n* = 4); reviews, letters, guidelines, editorials, conference abstracts, and poster presentations (*n*= 30); focused on glioma and metastasis differentiation (*n* = 12); focused on segmentation performance (*n* = 5); another imaging modality was used rather than MRI (*n* = 6); machine learning was used, not deep learning (*n* = 2).

Finally, 25 eligible studies were identified [14,25,26,27,28,29,30,31,32,33,34,35,36,37,38,39,40,41,42,43,44,45,46,47,48]. Due to a lack of sensitivity reporting, one study was included in the review but not in the quantitative analysis.

### 3.2. Quality Assessment

Table 1 shows a quality assessment summary of the included studies using the CLAIM. The mean CLAIM score of the four studies was 26.56 with a standard deviation (SD) of 3.19 (range, 18.00–31.00). The mean scores of the subsections of the CLAIM were 1.52 (SD = 0.71) for the title/abstract section, 2.00 (SD = 0.00) for the introduction section, 18.16 (SD = 2.25) for the methods section, 2.00 (SD = 0.82) for the results section, 1.96 (SD = 0.20) for the discussion section, and 0.92 (SD = 0.28) for the other information section.

**Figure 3:**
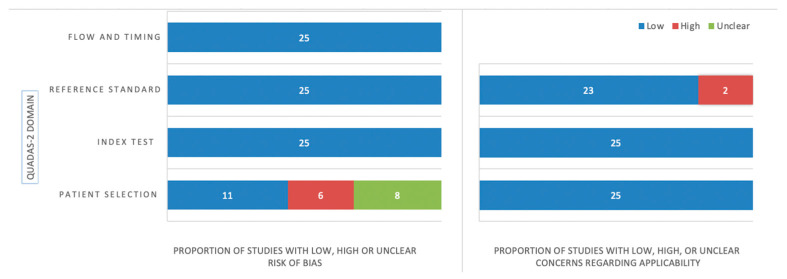
A summary of the Quality Assessment of Diagnostic Accuracy Studies-2 results.

Figure 3 illustrates a quality assessment summary of the included studies using the QUADAS-2 tool. In terms of patient selection, eight studies showed an unclear risk of bias because they did not specify the inclusion criteria for patient enrollment, or they did not report the excluded patients or exclusion criteria [26,30,33,34,35,41,45,47]. Six studies were found to have a high risk of bias due to patient exclusion based on lesion size or the number of lesions [25,29,31,32,37,38]. The inclusion and exclusion criteria of the studies can be found online in Supplementary Table S3. All included studies in the index test section were determined to have a low bias risk since the algorithm was blinded to the reference standard. Since the lesions were annotated on contrast-enhanced MRI manually or semi-manually with manual correction, which is the method of choice for assessing and delineating brain metastases, all studies in the reference standard section were considered to have a low risk of bias [5]. No risk of bias was found concerning the flow and timing. There were no concerns regarding the applicability of patient selection, and index tests. Two studies were found to have high concerns about the applicability of the reference test because they used 1.0 Tesla (T) scanners [36,40]. In current practice, scanners with magnets less than 1.5T are not recommended for BM detection [49].

### 3.3. Characteristics of Included Studies

The patient and study characteristics are shown in Table 2. All studies were conducted retrospectively. Five studies were multi-center studies [14,32,36,43,44], and the others were single-center. Four studies used cross-validation [30,31,38,43], and others used split training-test sets to evaluate their models. Delineation of brain metastases was done semi-automatically in one study to serve as a reference standard [25], and it was done manually in others. There were 6840, 1419, and 643 patients in the training and validation sets combined, test sets, and other sets, respectively. Ultimately, 25 studies included a total of 8902 patients. The number of metastatic lesions in the training sets was reported in 20 studies, totaling 31,530. Furthermore, the number of metastatic lesions in the test sets was documented in 18 studies, making a total of 5565. The total reported number of metastatic lesions was 40,654. Twenty-two studies included multiple primary tumor types, and three studies included only one primary tumor type. Two articles included only malignant melanoma patients, and one included non-small cell lung cancer (NSCLC) patients [30,36,40]. Table 2 also displays the means or medians of the volumes of the lesions or the longest diameter of the lesions.

A summary of the scanning details of the studies is shown in Table 3. Eleven studies included both 3.0T and 1.5T scanners [25,26,28,29,32,34,41,42,44,47,48]; two studies included only 1.5T scanners [27,30]; five studies included only 3.0T scanners [37,38,39,43,46]; two studies included 1.0T, 1.5T, and 3.0T scanners [36,40]. No information was found in five studies regarding their scanners’ magnets [14,31,33,35,45]. Ten studies used multiple MRI sequences in their models [14,26,27,32,36,38,39,40,41,42], whereas the remaining fifteen only used one. Two studies used 2D images [30,33], and seven used 2D and 3D images [14,26,27,36,38,40,41]. The remaining sixteen studies only used 3D images. All the studies included at least one sequence with contrast enhancement. With twenty studies, the most common included sequence was 3D contrast-enhanced T1 weighted imaging (WI). The slice thickness of images ranged from 0.43 mm to 7.22 mm (Table 3).

### 3.4. Deep Learning Algorithms

Table 4 provides a summary of detectability statistics, false-positive rates, and deep learning algorithm details. U-Net was used in ten studies [14,26,28,29,30,39,41,42,45,46], and DeepMedic was used in five [27,35,36,37,40]. Two studies implemented a single-shot detector [25,48]. Each of the following algorithms was used in a single study: You Only Look Once v3, fully convolution network, convolutional neural network (CNN), feature pyramid network, faster region-based CNN, input-level dropout model, Modified V-Net 3D, CropNet and noisy student. Data augmentation was not implemented in 6 studies [32,37,41,43,46,48], and in 19 studies, it was. False positive rates were reported in studies either per scan, per patient, per slice, per case, or per lesion. In two studies, false positive rates were not reported. Detailed information can be found in Table 4.

### 3.5. Assessment of Detectability Performance

In our study, we included the internal test set results or the cross-validation results in the pooled detectability analysis. If more than one algorithm was used in a study, we utilized the best algorithm in our pooled analysis. Furthermore, if more than one level of input was present, the one that yielded the most successful outcome was included in our study.

#### 3.5.1. Patient-Wise

**Figure 4:**
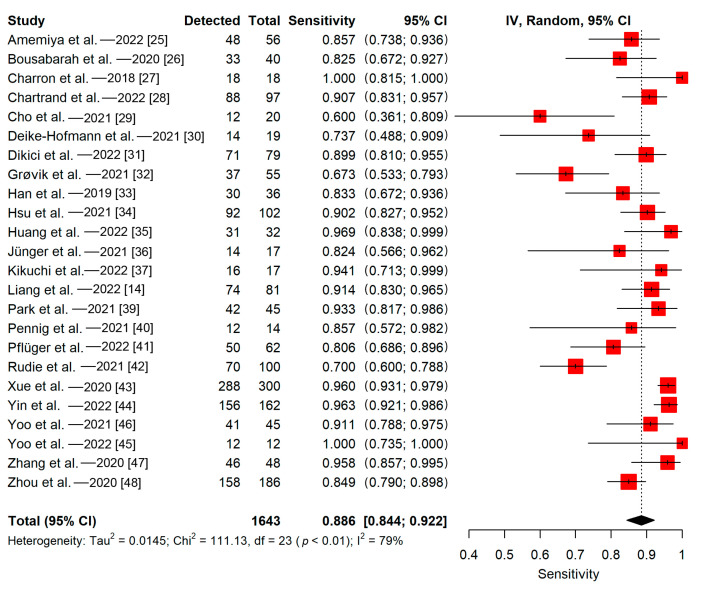
Forest plot of deep learning algorithms’ patient-wise detectability.

Twenty studies evaluated and reported their detectability lesion-wise, whereas the remaining four assessed and reported their detectability voxel-wise or patient-wise. The detectability of the 24 included studies ranged from 58% to 98%. The pooled proportion of detectability (patient-wise) of deep learning algorithms in all 24 included studies was 89% (95% CI, 84–92%) (Figure 4). The meta-regression did not find a significant impact of the training sample size on the sensitivity (*p* = 0.12). The Q-test indicated that heterogeneity was present across the studies (Q = 111.13, *p* < 0.01), and the Higgins I^2^ statistic showed the presence of high heterogeneity in detectability (I^2^ = 79%).

There was no statistically significant difference in performance between the groups in any subgroup analyses (*p*-values ranged from 0.08 to 0.69). Heterogeneity was moderate or high in subgroups that are separated based on the plurality of MRI sequences utilized (single MRI sequence; Q = 50.86, *p* < 0.01; I^2^ = 72%/multiple MRI sequences; Q = 33.09, *p* < 0.01; I^2^ = 76%), validation method (split training-test; Q = 83.18, *p* < 0.01; I^2^ = 76%/cross-validation; Q = 11.63, *p* < 0.01; I^2^ = 83%), study design (single-center; Q = 52.65, *p* < 0.01; I^2^ = 66%/multi-center; Q = 36.83, *p* < 0.01; I^2^ = 89%), and the calculation method of the reported sensitivity (lesion-wise; Q = 68.78, *p* < 0.01; I^2^ = 72%/others; Q = 37.60, *p* < 0.01; I^2^ = 92%). Subgroup analyses based on the plurality of the primary tumor type revealed a high heterogeneity in studies with multiple primary tumor types (Q = 105.92, *p* < 0.01; I^2^ = 81%). There was no evidence of heterogeneity in studies with a single primary tumor type (Q = 0.66, *p* = 0.72; I^2^ = 0%). Furthermore, subgroup analyses based on the dimension of the included images revealed high heterogeneity in studies with 3D images (Q = 94.2, *p* < 0.01; I^2^ = 84%). There was no evidence of heterogeneity in studies with a mixture of 2D and 3D images (Q = 9.64, *p* = 0.09; I^2^ = 48%) and studies with 2D images (Q = 0.72, *p* = 0.39; I^2^ = 0%). The meta-regression revealed that the heterogeneity not explained by the training sample size was significant, indicating that the training sample size did not influence heterogeneity significantly (*p* < 0.0001).

**Figure 5:**
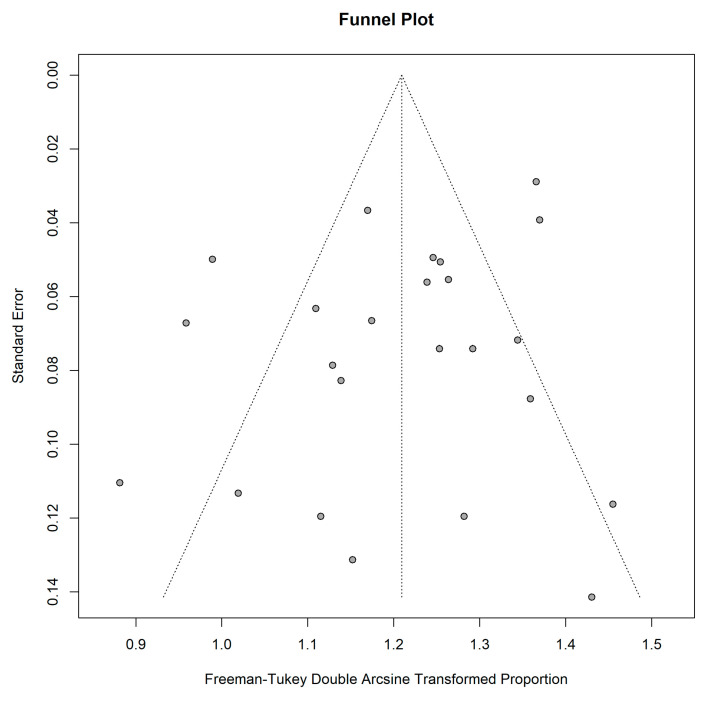
Funnel plot for studies included in the pooled analysis for patient-wise detectability.

The funnel plot was asymmetrical, indicating publication bias among the included studies (Figure 5). Furthermore, not all studies were plotted within the area under the curve of the pseudo-95% CI, showing a possible publication bias [50]. However, the Egger test did not indicate obvious publication bias (regression intercept = 1.32, *p* = 0.15).

#### 3.5.2. Lesion-Wise

**Figure 6:**
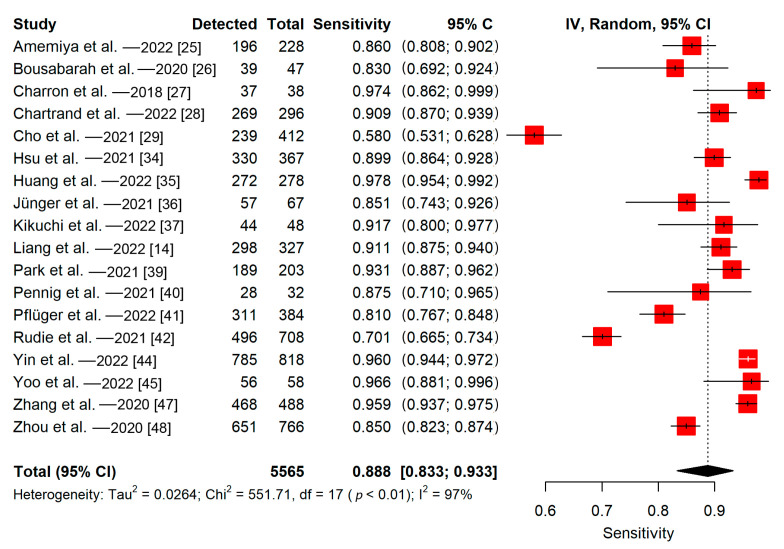
Forest plot of deep learning algorithms’ lesion-wise detectability.

Among 20 studies evaluated and reported their detectability lesion-wise, 18 of them reported the number of metastases in the test sets. All studies in this group used split training-test sets to evaluate their models. The detectability of the 18 included studies ranged from 58% to 98%. The pooled proportion of detectability of deep learning algorithms (lesion-wise) was 89% (95% CI, 83–93%) (Figure 6). The meta-regression did not find a significant impact of the training sample size on the sensitivity (*p* = 0.86). The Q-test indicated that heterogeneity was present across the studies (Q = 551.71, *p* < 0.01), and the Higgins I^2^ statistic showed the presence of high heterogeneity in detectability (I^2^ = 97%).

There was no statistically significant difference in performance between the groups in any subgroup analyses (*p*-values ranged from 0.26 to 0.68). Heterogeneity was high in subgroups that were separated based on the plurality of MRI sequences (single MRI sequence; Q = 370.72, *p* < 0.01; I^2^ = 98%/multiple MRI sequences; Q = 112.75, *p* < 0.01; I^2^ = 94%), study design (single-center; Q = 439.53, *p* < 0.01; I^2^ = 97%/multi-center; Q = 17.01, *p* < 0.01; I^2^ = 88%), and the dimension of the included images (3D images; Q = 529.39, *p* < 0.01; I^2^ = 98%/both 2D and 3D images; Q = 21.47, *p* < 0.01; I^2^ = 77%). Subgroup analyses based on the plurality of the primary tumor type revealed a high heterogeneity in studies with multiple primary tumor types (Q = 551.3, *p* < 0.01; I^2^ = 97%). There was no evidence of heterogeneity in studies with a single primary tumor type (Q = 0.06, *p* = 0.81; I^2^ = 0%). In this group, two subgroup analyses to investigate heterogeneity could not be carried out: based on validation method (all studies used split training-test method) and detection level (all studies reported lesion-wise sensitivity). The meta-regression showed that the heterogeneity not explained by the training sample size was significant, indicating that the training sample size had no significant effect on heterogeneity (*p* < 0.0001).

**Figure 7:**
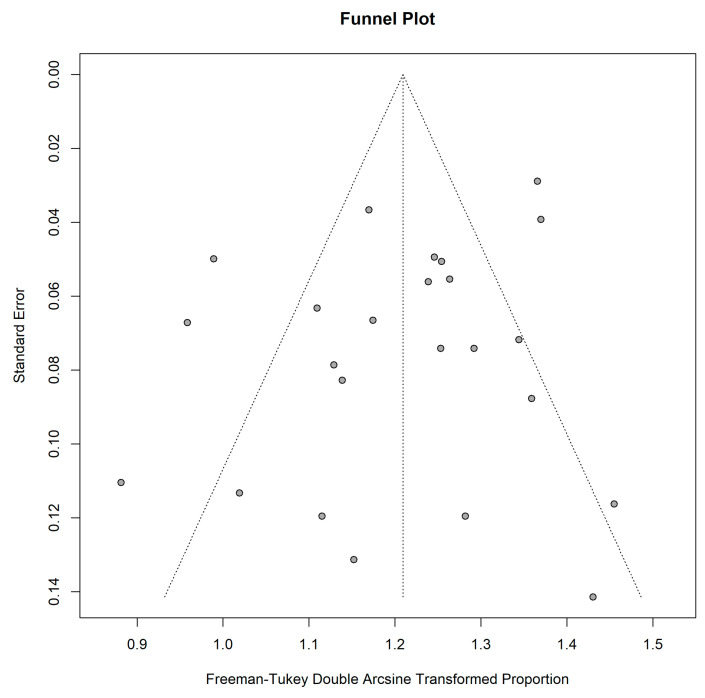
Funnel plot for studies included in the pooled analysis for lesion-wise detectability.

The asymmetrical funnel plot indicated publication bias among the included studies in this group (Figure 7). In addition, only some studies were plotted within the area under the curve of the pseudo-95% CI, indicating possible publication bias [50]. On the other hand, the Egger test revealed no obvious publication bias (regression intercept = 1.17, *p* = 0.66).

## 4. Discussion

BMs are ten times more common than primary malignant brain tumors, and patients with a history of BM should be followed up by imaging every three months and whenever clinically indicated [51,52]. Therefore, there is a massive demand for radiologists to detect and follow-up on these lesions. However, radiologists face several challenges in detecting BMs accurately. Among the challenges are a massive workload, difficulties in differentiation of BMs from noises and blood vessels, BMs with small sizes, significant variations in lesion shape and size among patients, weak signal intensities, and multiple locations and lesions in a patient [11]. As a result of these challenges, the use of deep learning methods in detecting BMs has recently increased in research. Because the recent meta-analysis by Cho et al. included just 12 studies, only 7 of which were deep learning studies, we decided to conduct a systematic review and assess the strength of the present evidence [11]. Their study also found a statistically significant difference in false positives between the deep learning and machine learning groups, necessitating the focused study on each group. Our analysis showed that studies with deep learning models using MR images perform well in detecting BMs, with a pooled sensitivity of 89% (95% CI, 84–92%). The detectability ranged from 58% to 98%.

The size of the lesions is an important determinant of the success of detecting BMs with deep learning algorithms [53]. For instance, in a study by Rudie et al., detection sensitivity for BMs smaller than 3 mm was only 14.6%, and sensitivity for BMs greater than 3 mm was 84.3% [42]. In another study, the detection sensitivity was reduced by 71% for BMs < 3 mm compared to all BMs [48]. Various approaches to solving this problem have been proposed in the literature. Amemiya et al. reinforced their SSD model for small lesion detections with feature fusion and managed to increase its sensitivity from 35.4% to 45.8% for lesions smaller than 3 mm [25]. In another study, Bousabarah et al. showed that the sensitivity of their models for small BMs ranged from 0.40 to 0.51 [26]. They trained another model on a subsample containing only small BMs (smaller than 0.4 mL), showing an increased sensitivity of 0.62–0.68 for small BMs. It is worth noting that this model’s sensitivity was 0.43–0.53 for all sizes of BMs. As stated, Dikici et al. used a sample of smaller BMs and acquired results similar to theirs [54]. Although training deep learning models with small BMs would help to improve sensitivity in detecting BMs, there are concerns when applying this method to a heterogeneous group of BMs. Furthermore, since a meta-analysis demonstrated that black blood images successfully detect small lesions [55], Park et al. added black blood images to their model with gradient echo sequence images and increased their sensitivity by 23.5% for detecting BMs < 3 mm [39]. Kottlors et al. also showed that smaller BMs (<5 mm) could be detected better with deep learning models using black blood images [38]. Yin et al. detected BMs < 3 mm with an impressive sensitivity of 89% by only using contrast-enhanced T1WI, and having a sensitivity of 95.8% in all sizes of BMs [44]. We believe, in part, this success was due to a large number of metastases in the cohort (11514 BMs) and a high proportion of small BMs (<5 mm, 58.6%) in their sample. Therefore, more models that are trained with real-world data representation and successful on both small and large lesions are required. Moreover, surgery or SRS planning scan with a higher spatial resolution can be more sensitive for detecting metastases than a conventional scan, revealing lesions not previously detected [56]. Therefore, utilizing MRI scanners with a higher spatial resolution might improve the efficacy of deep learning models, particularly for smaller lesions. Furthermore, we also analyzed the impact of training sample size on detectability, but no statistically significant effect was observed. Studies in the literature demonstrate that the success of deep learning models typically increases with sample size [57,58]. Although our results contradict this, one possible explanation could be that even though some papers have a small number of patients, the number of lesions in such a small number of patients can be quite high. For instance, Charron et al. included 164 patients with 374 metastatic lesions, whereas Deike-Hofmann et al. included 43 patients with 494 metastatic lesions. However, training sample size (number of lesions) on detectability in the group reporting sensitivity lesions-wise was not statistically significant either, indicating further research is needed.

Various medical imaging modalities have unique characteristics and different reactions to different tissues in the human body. Our review showed that ten studies used multiple MRI sequences in their models whereas the remaining fifteen only used one. Our meta-analysis showed that there was no statistically significant difference between the pooled proportion of detectability of studies with a single MRI sequence and the pooled proportion of detectability of studies with multiple MRI sequences. Park et al. combined and separately used two MRI sequences in their model [39]. Gradient echo sequence and black blood images showed 76.8% and 92.6% sensitivity, respectively. Their combination showed 93.1% sensitivity. There was a statistically significant difference between the models using only gradient echo sequence and combined sequences, but no difference between the black blood model and the combined model. This was also evident in the models’ sensitivity in lesions smaller than 3 mm, with the gradient echo sequence model, black blood model, and the combined model showing a sensitivity of 23.5%, 82.4%, and 82.4%, respectively. Charron et al. showed that the model combining different MRI modalities surpassed the model using single modalities in their study [27]. Contrary to these findings, our subgroup analyses showed no statistically significant difference between models using single or multiple MRI sequences. Therefore, additional research would be needed to determine which MRI scanners and scanner combinations would be the optimal strategy for automatically detecting brain metastases with deep learning. Furthermore, there was no statistically significant difference in the performance of models that included only 2D, 3D, or a combination of them; however, this comparison is not ideal due to the small number of studies that included only 2D images and studies that included both 2D and 3D images.

Most BMs are caused by lung cancer, breast cancer, melanoma, and renal cell carcinoma [2]. Our review showed that two articles included only malignant melanoma patients, and one included NSCLC patients. Others included BMs patients with any primary tumor. The appearance of BMs on MRI may vary between primary tumors, particularly in NSCLC [36,59]. Due to that, Jünger et al. stated that different deep learning models for different BMs originating from different primary tumors should be designed to obtain a satisfactory detection performance [36]. Although this may be helpful to increase the performance of the model detecting BMs originating from the particular primary tumor, the primary tumor is unknown in 5–40% of patients demonstrating the symptoms of BMs [60]. Furthermore, the primary tumor in up to 15% of BM patients cannot be identified [61]. Therefore, generalized deep learning models might be required to detect BMs with unknown primary tumors. Furthermore, we performed subgroup analyses to compare the performance of studies with a single primary tumor type and studies with multiple primary tumor types. There was no statistically significant difference in their performance; however, this comparison was not ideal due to the small number of studies with a single primary tumor type compared to studies with multiple primary tumor types. This is still an open research area, and further studies would be needed.

Besides sensitivity, false positives were the other important measure reported by the included studies. An important cause of false positives was the similarity between BMs and blood vessels, as both may present with a small focus of hyperintensity on a contrast-enhanced T1WI [46]. Several studies showed that most or at least some of their false positives were in and near vascular structures [25,32,37,39,44,46,47]. There have been several proposed solutions to this problem in the literature. Grøvik et al. hypothesized that adding other MRI sequences, such as diffusion-weighted MRI, may help deal with false positives [32]. Furthermore, black blood imaging can be helpful since it can suppress blood vessel signals, which is also increasingly applied in clinical practice [25,38,39,44]. It is worth noting that Park et al. demonstrated that when only black blood imaging is used, false positives are significantly higher than when black blood and gradient echo sequences images are used together [39]. Furthermore, Deike-Hofmann et al. demonstrated that including a pre-diagnosis scan in their model greatly reduced false positives, but including additional sequences contrarily decreased the specificity [30]. They hypothesized that this result was due to models interpreting that lesions that change over time are more likely to be BMs, whereas stable structures such as blood vessels are less likely, as humans do. Finally, skull stripping was another method that significantly reduced false positives, particularly extra-axial ones [14,25,42]. We were unable to conduct a pooled analysis of false positive rates in our study since included studies reported false positive rates differently.

Various deep learning algorithms were applied to MR images to detect BMs. Our review revealed that the most commonly used deep learning algorithm in BMs detection was U-Net, with different versions [62]. U-Net is an algorithm for semantic segmentation, also known as pixel-based classification. A contracting encoder and an expanding decoder comprise the U-Net. The expanding decoder creates the label map after the contracting encoder extracts low-level features. The second most commonly used deep learning algorithm was DeepMedic (Biomedical Image Analysis Group, Department of Computing, Imperial College London, London, UK) [63]. DeepMedic is built around a 3D deep CNN and a 3D conditional random field. Unsurprisingly, both algorithms are the most widely used since they are easily accessible. They are likely to be used more in the future, and there is room for research into combining these models in BMs detection [64]. The performance of the deep learning models was also compared. However, there was no statistically significant difference between the patient-wise sensitivity group and the lesion-wise sensitivity group (*p* = 0.25 and *p* = 0.26, respectively).

The performance of the deep learning models should be compared to the performance of radiologists in detecting BMs. Kikuchi et al. compared their model with twelve radiologists; seven were board-certified, and five were residents [37]. Their model exhibited higher sensitivity (91.7%) than the radiologists (88.7 ± 3.7%), however the article did not compare how the algorithms performed compared to faculty versus trainees, and the lumping of the trainees with the faculty may explain the overall lower sensitivity of the radiologists. Rudie et al. compared the performance of two initial manual annotations with the deep learning model [42]. Contrary to the findings of the aforementioned study, the detection sensitivity was higher and statistically significant for radiologists than for the deep learning model for metastases smaller than 3 mm (63.2% versus 14.7%) and metastases between 3 and 6 mm (90.8% versus 76.7%). Liang et al. discovered that their deep learning model detected 2% of BMs that were overlooked during the manual annotation, even though the manual annotations were checked by two investigators and reevaluated by a senior radiation oncologist [14]. In addition, Yin et al. showed that with the assistance of the deep learning model, readers’ mean sensitivity increased by 21% [44]. Another meta-analysis that compared deep learning models’ performance with healthcare professionals in detecting diseases from medical images found that the diagnostic performance of deep learning models and healthcare professionals was equivalent [65]. We believe these results are auspicious, and although completely automated models cannot be fully implemented into clinical practice today, they may serve as a robust assistant for radiologists. We could not conduct a pooled analysis to compare deep learning models and radiologists in our study due to the small number of studies reporting radiologists’ sensitivity. The literature is lacking in this regard, and more papers comparing the two groups are needed, especially for small, difficult to detect lesions.

Our study was not without limitations. The main limitation was the lack of pooled false positive rate analysis since there was no uniform unit of reporting them in the included studies. Studies reported false positives in per-patient or per-scan or per-lesion. Second, subgroup analyses based on different MRI scanners and slice thicknesses were not possible because the results for different scanners and slice thicknesses were not included in any of the included studies. Furthermore, study heterogeneity was high in our analyses; but it was commonly observed in meta-analyses on imaging-based deep learning studies [13,66,67,68,69]. In addition, in our study, the subgroups with no evidence of heterogeneity included very few studies. However, it is known that Q test has inadequate power to detect true heterogeneity when the meta-analysis includes a small number of studies [70]. Therefore, it is likely that heterogeneity is not due to subgroups. It is worth noting that, even though it is not a limitation of our study, our quality assessment showed that reporting was poor in some studies.

## 5. Conclusions

Our study revealed that deep learning algorithms effectively detect BMs with a pooled sensitivity of 89%. In the future, deep learning studies should adhere to CLAIM and QUADAS-2 checklists more strictly. Uniform reporting standards that clearly explain the results of deep learning models in BMs detection are needed. Since all the included studies were conducted retrospectively, there is a need for additional large-scale prospective studies.

## Figures and Tables

**Table 1:** The Checklist for Artificial Intelligence in Medical Imaging scores.

Study	Title/Abstract Score (*n/*2)	Introduction Score (*n/*2)	Methods Score (*n/*28)	Results Score (*n/*5)	Discussion Score (*n/*2)	Other Information Score (*n/*3)	Total Score (*n/*42)
Amemiya et al.—2022 [25]	2	2	21	3	2	1	31
Bousabarah et al.—2020 [26]	2	2	18	2	2	1	27
Charron et al.—2018 [27]	1	2	17	0	2	1	23
Chartrand et al.—2022 [28]	2	2	18	1	2	1	26
Cho et al.—2021 [29]	2	2	19	2	2	1	28
Deike-Hofmann et al.—2021 [30]	1	2	17	2	2	1	25
Dikici et al.—2022 [31]	0	2	17	2	2	1	24
Grøvik et al.—2021 [32]	1	2	22	3	2	1	31
Han et al.—2019 [33]	0	2	13	1	1	1	18
Hsu et al.—2021 [34]	0	2	19	2	2	1	26
Huang et al.—2022 [35]	2	2	16	2	2	0	24
Jünger et al.—2021 [36]	2	2	19	2	2	1	28
Kikuchi et al.—2022 [37]	2	2	14	3	2	1	24
Kottlors et al.—2021 [38]	1	2	14	2	2	1	22
Liang et al.—2022 [14]	2	2	19	2	2	1	28
Park et al.—2021 [39]	2	2	18	3	2	1	28
Pennig et al.—2021 [40]	2	2	18	1	2	1	26
Pflüger et al.—2022 [41]	2	2	21	3	2	1	31
Rudie et al.—2021 [42]	2	2	21	2	2	1	30
Xue et al.—2020 [43]	2	2	20	3	2	1	30
Yin et al.—2022 [44]	2	2	20	3	2	1	30
Yoo et al.—2022 [45]	1	2	18	1	2	1	25
Yoo et al.—2021 [46]	2	2	17	1	2	0	24
Zhang et al.—2020 [47]	2	2	19	2	2	1	28
Zhou et al.—2020 [48]	1	2	19	2	2	1	27

**Table 2:** The patient and study characteristics.

Study	Year	Design	No of Patients in the Training Set (M:F)	No of Patients in the Validation Set (M:F)	No of Patients in the Test Set (M:F)	No of Patients in the Other Sets (M:F)	No of Metastatic Lesions (Training/Validation/Test/Other Sets)	Mean or Median—Whole Volume or Longest Diameter of Lesions (Training/Validation/Test/Other Sets)	Reference Standard	Validation Method	Primary Tumor
Amemiya et al. [25]	2022	Single-center	178 (96:82)	NA	56 (30:26)	NA	1249/NA/228/NA	4.1/NA/10.4/NA mm—mean	Semi-automatic	Split training-test	Multiple
Bousabarah et al. [26]	2020	Single-center	469 (244:225)	NA	40 (26:14)	NA	524/NA/47/NA	1.29/NA/1.92/NA cm^3^—mean	Manual	Split training-test	Multiple
Charron et al. [27]	2018	Single-center	164 (NR)	NA	18 (NR)	NA	374/NA/38/NA	8.1 mm (whole sample)—mean	Manual	Split training-test	Multiple
Chartrand et al. [28]	2022	Single-center	383 (NR)	50 (NR)	97 (NR) ^1^	NA	1460/NR/296/NA ^2^	NR/NR/NR/NA	Manual	Split training-test	Multiple
Cho et al. [29]	2021	Single-center	127 (61:66)	NA	20 (12:8)	47 (25:22)	1298/NA/412/NR	6.5/NA/6/NR mm—median	Manual	Split training-test	Multiple
Deike-Hofmann et al. [30]	2021	Single-center	43 (35:8)	NA	NA	NA	494/NA/NA/NA	4.2/NA/NA/NA mm—median	Manual	Cross-validation	Single/Malignant melanoma
Dikici et al. [31]	2022	Single-center	158 (M:F = 0.89) ^3^	NA	NA	NA	932/NA/NA/NA	5.45/NA/NA/NA mm—mean	Manual	Cross-validation	Multiple
Grøvik et al. [32]	2021	Multi-center	100 (29:71)	10 (NR) ^4^	55 (NR)	NA	NR/NR/NR/NA	NR/NR/NR/NA	Manual	Split training-test	Multiple
Han et al. [33]	2019	Single-center	126 (NR) ^5^	18 (NR)	36 (NR)	NA	NR/NR/NR/NA	NR/NR/NR/NA	Manual	Split training-test	Multiple
Hsu et al. [34]	2021	Single-center	409 (NR)	NA	102 (NR)	NA	1345/NA/367/NA	NR/NA/NR/NA	Manual	Split training-test	Multiple
Huang et al. [35]	2022	Single-center	135 (NR)	9 (NR)	32 (NR)	NA	1503/NR/278/NA	NR/NR/NR/NA	Manual	Split training-test	Multiple
Jünger et al. [36]	2021	Multi-center ^6^	66 (24:42)	NA	17 (6:11)	15 (5:10)	248/NA/67/0	0.99/NA/0.96/NA cm^3^—mean	Manual	Split training-test	Single/NSCLC
Kikuchi et al. [37]	2022	Single-center	50 (30:20)	NA	34 (16:18) ^7^	NA	165/NA/48/NA	4/NA/2.9/NA mm—median	Manual	Split training-test	Multiple
Kottlors et al. ^8^ [38]	2021	Single-center	85 (52.3%:47.7%) ^9^	NA	NA	NA	47/NA/NA/NA	NR/NA/NA/NA	Manual	Cross-validation	Multiple
Liang et al. [14]	2022	Multi-center	326 (127:150) ^10^	NA	81 (31:50)	NA	1284/NA/327/NA	15.9/NA/17.6/NA mm—median	Manual	Split training-test	Multiple
Park et al. [39]	2021	Single-center	188 (98:90)	NA	94 (55:39) ^11^	NA	917/NA/203/NA	1.6/NA/1.9/NA cm^3^—mean	Manual	Split training-test	Multiple
Pennig et al. [40]	2021	Single-center	55 (?) ^12^	NA	14 (NR)	NA	103/NA/32/NA	2.6/NA/1/NA cm^3^—mean	Manual	Split training-test	Single/Malignant melanoma
Pflüger et al. [41]	2022	Single-center	246 (134:112)	NA	62 (29:33)	30 (15:15)	1682/NA/384/155	1.23/NA/1.24/1.03 cm^3^—mean	Manual	Split training-test	Multiple
Rudie et al. [42]	2021	Single-center	313 (127:186)	NA	100 (48:52)	NA	4494/NA/708/NA	0.57/NA/0.50/NA cm^3^—mean	Manual	Split training-test	Multiple
Xue et al. [43]	2020	Multi-center	1201 (684:517)	NA	NA	251 (236:215)	NR/NA/NA/NR	4.01/NA/NA/NR cm^3^—mean	Manual	Cross-validation	Multiple
Yin et al. ^13^ [44]	2022	Multi-center	680 (374:306)	NA	270 (144:2126)	300 (161:139)	9630/NA/818/1066	5.5/NA/7.5/5.8 mm—mean	Manual	Split training-test	Multiple
Yoo et al. [45]	2022	Single-center	53 (29:24)	NA	12 (6:6)	NA	545/NA/58/NA	0.592/NA/0.158/NA cm^3^—mean	Manual	Split training-test	Multiple
Yoo et al. [46]	2021	Single-center	341 (NR)	36 (NR)	45 (NR)	NA	NR/NR/NR/NA ^14^	NR/NR/NA/4.17 cm^3^—mean	Manual	Split training-test	Multiple
Zhang et al. [47]	2020	Single-center	73 (NR) ^15^	NA	48 (NR)	NA	1565/NA/488/NA	NR/NA/NR/NA	Manual	Split training-test	Multiple
Zhou et al. [48]	2020	Single-center	748 (NR) ^16^	NA	186 (NR)	NA	3131/NA/766/NA	NR/NA/NR/NA ^17^	Manual	Split training-test	Multiple

Abbreviations: No, number; M, male; F, female; NA, not applicable; mm, millimeter; cm^3^, cubic centimeter; NR, not reported. ^1^ The total dataset contains 291 females and 239 males. ^2^ The combined training and validation sets contain 1460 lesions. ^3^ Unlabeled data contained 867 patients. ^4^ Validation and testing sets included 35 female and 30 male patients combined. ^5^ Synthetic images are not included in our study. ^6^ The authors stated, “this study included imaging data from referring institutions, it does not approximate to a true multicenter approach” in the limitations. ^7^ The test group included 17 patients with brain metastases and 17 without enhancing lesions. ^8^ This study is not included in the quantitative analysis since they did not report the sensitivity. ^9^ Fifty-nine patients do not have brain metastases. ^10^ Due to image anonymization, gender information was missing in 49 patients. ^11^ The test set included 45 patients with brain metastases and 49 patients without. ^12^ The total dataset contains 30 females and 39 males. ^13^ The sets included 269, 108, and 529 patients without brain metastases, respectively. ^14^ The whole dataset included 878 metastatic lesions. ^15^ The total dataset contains 46 males and 75 females. ^16^ The total dataset contains 460 males and 474 females. ^17^ The mean size of the metastases in the entire dataset was 9 mm.

**Table 3:** Scanning characteristics.

Study	Slice Thickness	Scanning Sequences	Scanner	Tesla
Amemiya et al.—2022 [25]	1 mm	3D CE T1WI	SIEMENS MAGNETOM Skyra; SIEMENS MAGNETOM Avanto; GE Signa EXCITE HDxt x2; GE Premier; GE Signa EXCITE HDxt; GE Signa EXCITE HD; Toshiba Excelart Vantage; Phillips Ingenia CX	3T; 1.5T; 3T; 3T; 1.5T; 1.5T; 1.5T; 3T
Bousabarah et al.—2020 [26]	NR	2D/3D CE T1WI, 2D/3D T2WI, 2D/3D FLAIR	Philips Ingenia; Philips Ingenia; Philips Archieva; Philips Intera	3T; 1.5T; 3T; 1.5T
Charron et al.—2018 [27]	1.02 mm	3D CE T1WI, 2D FLAIR	NR	1.5T
Chartrand et al.—2022 [28]	1, 1.50 or 2 mm	3D CE T1WI	Philips Ingenia Elition; Philips Achieva; Siemens MAGNETOM^®^ Aera; Siemens MAGNETOM^®^ Avanto fit; GE SIGNA™ Explorer; GE Optima™ MR450w GEM; GE Discovery™ MR750; Philips Intera; Siemens MAGNETOM Skyra	3T; 3T; 1.5T; 1.5T; 1.5T; 1.5T; 3T; 1.5T; 3T
Cho et al.—2021 [29]	1 mm	3D CE T1WI	Philips Intera; Philips Achieva; Philips Ingenia; SIEMENS Verio	1.5T; 3T; 3T; 3T
Deike-Hofmann et al.—2021 [30]	4 mm	2D CE T1WI	SIEMENS MAGNETOM Symphony	1.5T
Dikici et al.—2022 [31]	NR	3D CE T1WI	NR	NR
Grøvik et al.—2021 [32]	0.90 to 1.60 mm	3D T1WI, 3D CE T1WI, 3D Black Blood, 3D CE FLAIR	GE TwinSpeed; GE SIGNA Explorer; GE SIGNA Architect; GE Discovery 750 and 750w; SIEMENS Skyra	1.5T; 1.5T; 3T; 3T; 3T
Han et al.—2019 [33]	NR	2D CE T1WI	NR	NR
Hsu et al.—2021 [34]	1 to 1.98 mm	3D CE T1WI	GE Discovery MR750w; GE Optima MR450w; GE Signa PET/MR; GE Signa HDxt; GE Signa Architect; GE Signa Artist; GE Signa Excite; Philips Ingenia; SIEMENS Aera	3T; 1.5T; 3T; 1.5T; 3T; 1.5T; 1.5T; 3T; 1.5T
Huang et al.—2022 [35]	1 mm	3D CE T1WI	NR	NR
Jünger et al.—2021 [36]	2 to 6 mm	2D/3D T1WI, 2D/3D CE T1WI, 2D/3D T2WI, 2D/3D FLAIR	Philips Achieva; GE Optima; Philips Ingenia; Philips Intera; Philips Panorama; Siemens Aera; Siemens Amira; Siemens Avanto; Siemens Espree; Siemens Skyra; Siemens Symphony; Siemens Prisma	1T or 1.5T or 3T
Kikuchi et al.—2022 [37]	2 mm	3D Black Blood	Philips Achieva; Philips Ingenia	3T; 3T
Kottlors et al.—2021 [38]	1 mm, 5 mm	2D CE T1WI; 3D Black Blood	Philips Ingenia	3T
Liang et al.—2022 [14]	0.43 to 7.22 mm	2D/3D CE T1WI; 2D/3D FLAIR	NR (The MR images were acquired on 14 types of scanners from 4 major vendors—Siemens, GE, Philips, and Toshiba).	NR
Park et al.—2021 [39]	1 mm	3D Black Blood, 3D GRE	Philips Achieva; Philips Ingenia; Philips Ingenia CX; Philips Ingenia Elition X	3T; 3T; 3T; 3T
Pennig et al.—2021 [40]	2.30 to 5.20 mm	2D T1WI, 2D/3D CE T1WI, 2D T2WI, 2D FLAIR	Philips Achieva; Philips Gyroscan; Philips Ingenia; Philips Intera; Philips Panorama; SIEMENS Avanto; SIEMENS Biograph; GE Optima; GE Genesis Signa	1T or 1.5T or 3T
Pflüger et al.—2022 [41]	1 to 5 mm	2D T1WI, 3D CE T1WI, 2D FLAIR	SIEMENS Magnetom Verio; SIEMENS Skyra; SIEMENS Trio TIM; SIEMENS Magnetom Avanto	3T; 3T; 3T; 1.5T
Rudie et al.—2021 [42]	1.50 mm	3D T1WI, 3D CE T1WI	GE Signa HDxt; Philips Achieva; GE Discovery MR750	1.5T; 1.5T; 3T
Xue et al.—2020 [43]	1.50 mm	3D CE T1WI	SIEMENS MAGNETOM Skyra	3T
Yin et al.—2022 [44]	1 mm	3D CE T1WI	MAGNETOM Aera; Discovery MR750; Discovery MR750W; SIGNA Pioneer; SIGNA Premier; SIGNA Architect; Ingenia CX; MAGNETOM Trio Tim; MAGNETOM Prisma; uMR560; uMR780; uMR790; Optima MR360; MAGNETOM Skyra; MAGNETOM Verio	1.5T; 3T; 3T; 3T; 3T; 3T; 3T; 3T; 3T; 3T; 3T; 3T; 1.5T; 3T; 3T
Yoo et al.—2022 [45]	1 mm	3D CE T1WI	NR	NR
Yoo et al.—2021 [46]	0.90 mm	3D CE T1WI	SIEMENS MAGNETOM Skyra	3T
Zhang et al.—2020 [47]	0.89 to 3.84 mm	3D CE T1WI	NR	1.5T or 3T
Zhou et al.—2020 [48]	1 mm	3D CE T1WI	GE Signa HDxt; GE Discovery MR750w	1.5T; 3T

Abbreviations: CE, contrast-enhanced; WI, weighted imaging; GE, General Electric; T, Tesla; FLAIR, fluid attenuated inversion recovery; GRE, gradient echo.

**Table 4:** Algorithms and statistics.

Study	Detectability/Test Level	False Positive Rate	DL Algorithm	Data Augmentation
Studies with lesion-wise sensitivity reporting				
Amemiya et al.—2022 [25]	0.86/lesion-wise	4.3 per scan	Single-shot detector	Yes
Bousabarah et al.—2020 [26]	0.82/lesion-wise	0.35 per lesion	Conventional U-Net and modified U-Net	Yes
Charron et al.—2018 [27]	0.98/lesion-wise	14.2 per patient	DeepMedic	Yes
Chartrand et al.—2022 [28]	0.909/lesion-wise	0.66 per scan	3D U-Net	Yes
Cho et al.—2021 [29]	0.58/lesion-wise	2.5 per scan	3D U-Net	Yes
Han et al.—2019 [33]	0.83/lesion-wise	3.59 per slice	You Only Look Once v3	Yes
Hsu et al.—2021 [34]	0.90/lesion-wise	3.4 per patient	Modified V-Net 3D	Yes
Huang et al.—2022 [35]	0.975/lesion-wise	6.97 per patient *	DeepMedic	Yes
Jünger et al.—2021 [36]	0.851/lesion-wise	1.5 per scan	DeepMedic	Yes
Kikuchi et al.—2022 [37]	0.917/lesion-wise	1.5 per case	DeepMedic	No
Liang et al.—2022 [14]	0.91/lesion-wise	1.7 per scan	U-Net	Yes
Park et al.—2021 [39]	0.931/lesion-wise	0.59 per patient	3D U-Net	Yes
Pennig et al.—2021 [40]	0.88/lesion-wise	0.71 per scan	DeepMedic	Yes
Pflüger et al.—2022 [41]	0.81/lesion-wise	0.87 per scan	nnU-Net	No
Rudie et al.—2021 [42]	0.70/lesion-wise	0.46 per scan	3D U-Net	Yes
Yin et al.—2022 [43]	0.958/lesion-wise	0.39 per scan	FPN	Yes
Yoo et al.—2022 [45]	0.966/lesion-wise	1.25 per patient	2D U-Net	Yes
Yoo et al.—2021 [46]	0.91/lesion-wise	7.67 per patient	3D U-Net	No
Zhang et al.—2020 [47]	0.956/lesion-wise	19.9 per scan	Faster R-CNN *	Yes
Zhou et al.—2020 [48]	0.85/lesion-wise	3 per patient	Single-shot detector	No
Studies with patient-wise or voxel-wise sensitivity reporting				
Deike-Hofmann et al.—2021 [30]	0.727/patient-wise	6.6 per case	U-Net	Yes
Dikici et al.—2022 [31]	0.9/patient-wise	8.44 per patient	CropNet and noisy student	Yes
Grovik et al.—2021 [32]	0.671/voxel-wise	12.3 per lesion	Input-level dropout model	No
Xue et al.—2019 [43]	0.96/voxel-wise	Not reported	3D FCN	No
Studies not included in the quantitative analysis				
Kottlors et al.—2021 [38]	NR	NR	CNN	Yes

* Calculated using the study’s presented data.

## Supplementary Materials

The following supporting information can be downloaded at: https://www.mdpi.com/article/10.3390/cancers15020334/s1, Table S1: PRISMA-DTA Abstract Checklist; Table S2: PRISMA-DTA Checklist; Table S3: Inclusion and exclusion criteria.
