# Supplementary material for: Deep Learning for Detecting Brain Metastases on MRI: A Systematic Review and Meta-Analysis
Source: Cancers (Basel). 2023 Jan 4;15(2):334. doi: 10.3390/cancers15020334 (PMC9857123; doi:10.3390/cancers15020334)
Supplement: Supplementary file 1 [file cancers-15-00334-s001.zip › cancers-2122622-supplementary.pdf]

Supplementary Materials: Deep Learning for Detecting Brain Metastases on MRI: A Systematic Review and Meta-analysis

Burak B. Ozkara, Melissa M. Chen, Christian Federau, Mert Karabacak, Tina M. Briere, Jing Li and Max Wintermark

Table S1. PRISMA-DTA Abstract Checklist.

Section/Topic	Number #	PRISMA-DTA for Abstracts Checklist Item	Reported on Page #
		TITLE and PURPOSE	
Title	1	Identify the report as a systematic review (+/- meta-analysis) of diagnostic test accuracy (DTA) studies.	1
Objectives	2	Indicate the research question, including components such as participants, index test, and target conditions.	1
		METHODS	
Eligibility criteria	3	Include study characteristics used as criteria for eligibility.	1
Information sources	4	List the key databases searched and the search dates.	1
Risk of bias & applicability	5	Indicate the methods of assessing risk of bias and applicability.	1
Synthesis of results	A1	Indicate the methods for the data synthesis.	1
		RESULTS	
Included studies	6	Indicate the number and type of included studies and the participants and relevant characteristics of the studies (including the reference standard).	1
Synthesis of results	7	Include the results for the analysis of diagnostic accuracy, preferably indicating the number of studies and participants. Describe test accuracy including variability; if meta-analysis was done, include summary results and confidence intervals.	1
		DISCUSSION	
Strengths and limitations	9	Provide a brief summary of the strengths and limitations of the evidence	1
Interpretation	10	Provide a general interpretation of the results and the important implications.	1
		OTHER	
Funding	11	Indicate the primary source of funding for the review.	N/A
Registration	12	Provide the registration number and the registry name	N/A

Adapted From: McInnes MDF, Moher D, Thombs BD, McGrath TA, Bossuyt PM, The PRISMA-DTA Group (2018). Preferred Reporting Items for a Systematic Review and Meta-analysis of Diagnostic Test Accuracy Studies: The PRISMA-DTA Statement. JAMA. 2018 Jan 23;319(4):388-396. doi: 10.1001/jama.2017.19163.For more information, visit: www.prisma-statement.org.

Table S2. PRISMA-DTA Checklist.

Section/Topic	Number #	PRISMA-DTA Checklist Item	Reported on Page #
		TITLE / ABSTRACT	
Title	1	Identify the report as a systematic review (+/- meta-analysis) of diagnostic test accuracy (DTA) studies.	1
Abstract	2	Abstract: See PRISMA-DTA for abstracts.	
		INTRODUCTION	
Rationale	3	Describe the rationale for the review in the context of what is already known.	2
Clinical role of index test	D1	State the scientific and clinical background, including the intended use and clinical role of the index test, and if applicable, the rationale for minimally acceptable test accuracy (or minimum difference in accuracy for comparative design).	2
Objectives	4	Provide an explicit statement of question(s) being addressed in terms of participants, index test(s), and target condition(s).	2
		METHODS	

Protocol and registration	5	Indicate if a review protocol exists, if and where it can be accessed (e.g., Web address), and, if available, provide registration information including registration number.	N/A
Eligibility criteria	6	Specify study characteristics (participants, setting, index test(s), reference standard(s), target condition(s), and study design) and report characteristics (e.g., years considered, language, publication status) used as criteria for eligibility, giving rationale.	3
Information sources	7	Describe all information sources (e.g., databases with dates of coverage, contact with study authors to identify additional studies) in the search and date last searched.	3
Search	8	Present full search strategies for all electronic databases and other sources searched, including any limits used, such that they could be repeated.	3
Study selection	9	State the process for selecting studies (i.e., screening, eligibility, included in systematic review, and, if applicable, included in the meta-analysis).	3
Data collection process	10	Describe method of data extraction from reports (e.g., piloted forms, independently, in duplicate) and any processes for obtaining and confirming data from investigators.	3
Definitions for data extraction	11	Provide definitions used in data extraction and classifications of target condition(s), index test(s), reference standard(s) and other characteristics (e.g. study design, clinical setting).	3
Risk of bias and applicability	12	Describe methods used for assessing risk of bias in individual studies and concerns regarding the applicability to the review question.	3
Diagnostic accuracy measures	13	State the principal diagnostic accuracy measure(s) reported (e.g. sensitivity, specificity) and state the unit of assessment (e.g. per-patient, per-lesion).	4
Synthesis of results	14	Describe methods of handling data, combining results of studies and describing variability between studies. This could include, but is not limited to: a) handling of multiple definitions of target condition. b) handling of multiple thresholds of test positivity, c) handling multiple index test readers, d) handling of indeterminate test results, e) grouping and comparing tests, f) handling of different reference standards	4
Meta-analysis	D2	Report the statistical methods used for meta-analyses, if performed.	4
Additional analyses	16	Describe methods of additional analyses (e.g., sensitivity or subgroup analyses, meta-regression), if done, indicating which were pre-specified.	4
		RESULTS	
Study selection	17	Provide numbers of studies screened, assessed for eligibility, included in the review (and included in meta-analysis, if applicable) with reasons for exclusions at each stage, ideally with a flow diagram.	5
Study characteristics	18	For each included study provide citations and present key characteristics including: a) participant characteristics (presentation, prior testing), b) clinical setting, c) study design, d) target condition definition, e) index test, f) reference standard, g) sample size, h) funding sources	7-11
Risk of bias and applicability	19	Present evaluation of risk of bias and concerns regarding applicability for each study.	5-7
Results of individual studies	20	For each analysis in each study (e.g. unique combination of index test, reference standard, and positivity threshold) report 2x2 data (TP, FP, FN, TN) with estimates of diagnostic accuracy and confidence intervals, ideally with a forest or receiver operator characteristic (ROC) plot.	N/A
Synthesis of results	21	Describe test accuracy, including variability; if meta-analysis was done, include results and confidence intervals.	15-17
Additional analysis	23	Give results of additional analyses, if done (e.g., sensitivity or subgroup analyses, meta-regression; analysis of index test: failure rates, proportion of inconclusive results, adverse events).	15-17
		DISCUSSION	
Summary of evidence	24	Summarize the main findings including the strength of evidence.	18

Limitations	25	Discuss limitations from included studies (e.g. risk of bias and concerns regarding applicability) and from the review process (e.g. incomplete retrieval of identified research).	21
Conclusions	26	Provide a general interpretation of the results in the context of other evidence. Discuss implications for future research and clinical practice (e.g. the intended use and clinical role of the index test).	21
		FUNDING	
Funding	27	For the systematic review, describe the sources of funding and other support and the role of the funders.	21

Adapted From: McInnes MDF, Moher D, Thombs BD, McGrath TA, Bossuyt PM, The PRISMA-DTA Group (2018). Preferred Reporting Items for a Systematic Review and Meta-analysis of Diagnostic Test Accuracy Studies: The PRISMA-DTA Statement. JAMA. 2018 Jan 23;319(4):388-396. doi: 10.1001/jama.2017.19163.For more information, visit: www.prisma-statement.org.

Table S3. Inclusion and exclusion criteria.

Study—Year	Inclusion Criteria	Exclusion Criteria
Amemiya et al.—2022 [25]	I.Patients with brain metastasis who has no prior open surgery history<br>II.No primary brain cancers<br>.CE T1WI reconstructed to 1 mm are available	I.Scans containing more than 100 lesions<br>II.Images with severe artifacts
Bousabarah et al.—2020 [26]	I.Stereotactic radiosurgery candidates with brain metastasis	I.Unavailable sequences
Charron et al.—2018 [27]	I.Patients with brain metastasis who has no prior surgery history<br>II.No prior radiation therapy<br>.Same MRI machine used a week before radiotherapy treatment	Not reported
Chartrand et al.—2022 [28]	I.3D CE T1WI MRI with at least one brain metastasis<br>II.Patient treated with radiotherapy<br>I.Scans carried out between 2015 and 2020	I.Motion artifacts or low image quality<br>II.Presence of leptomeningeal lesions<br>III.Lesions outside the brain
Cho et al.—2021 [29]	I.Patients with systemic cancer and underwent brain metastasis MRI workup protocol	I.History of primary brain cancers<br>I.Presence of bone, dura, or skin metastasis, or questionable lesions for leptomeningeal seeding<br>I.Existence of other pathological situations (i.e., meningioma, vestibular schwannoma, pituitary adenoma, cavernous malformation, or hemorrhagic infarction)<br>IV.Presence of equivocal nodule(s)<br>V.Presence of disproportionate artifacts or poor image quality<br>I.Presence of more than 50 metastatic nodules<br>VII.Absence of brain metastasis
Deike-Hofmann et al.—2021 [30]	.Patients with malignant melanoma spread to cranium<br>I.Availability of the native T1WI, CE T1WI, T2WI, and FLAIR MRI sequences	Not reported
Dikici et al.—2022 [31]	Patients with brain metastasis and available 3D CE T1WI	.Patients with metastases larger than 15 mm<br>II.Patients with primary brain neoplasms, central nervous system lymphoma, extraaxial disease, leptomeningeal disease, or equivocally enhancing foci

Grøvik et al.—2021—Training sample [32]	I.The presence of known or possible metastatic disease<br>II.No prior surgical or radiation therapy<br>.The availability of all required MR imaging sequences<br>IV.Patients with ≥1 metastatic lesion	Not reported
Grøvik et al.—2021—Validation and test sample [32]	.Patients received stereotactic radiosurgery for at least one brain metastasis that measured at least 5 mm in one direction<br>I.Patients who are untreated or progressive after systemic or local therapy<br>Patients who have confirmed non-small cell lung cancer or malignant melanoma<br>IV.≥18 years of age<br>.Patients who have an Eastern Cooperative Oncology Group performance status score ≤1<br>VI.Patients who have a life expectancy >6 weeks	Not reported
Han et al.—2019 [33]	.Brain metastatic cancer cases with CE T1WI	Not reported
Hsu et al.—2021 [34]	.Patients who underwent stereotactic radiosurgery for brain metastases with CE 3D spoiled-gradient MR sequence	Not reported
Huang et al.—2022 [35]	.Brain metastatic cancer cases with CE T1WI	Not reported
Jünger et al.—2021 [36]	.MRI scans at diagnosis of brain metastases<br>I.Metastasis specific therapy after diagnosis<br>II.Complete multiparametric MRI images	I.Severe MRI artifacts<br>II.Insufficient application of contrast agent<br>.Incomplete coverage of the brain in one or more MRI sequences
Kikuchi et al.—2022 [37]	.Brain metastasis patients with VISIBLE imaging	I.No enhancement of intraparenchymal lesions<br>I.No follow-up examination with VISIBLE<br>III.No change in lesion size on follow-up<br>IV.Artifacts<br>V.Extra-axial tumors or infarctions<br>VI.More than eleven lesions
Kottlors et al.—2021 [38]	I.Patients receiving screening MRI for brain metastases<br>II.Patients received both, BB, and conventional CE T1WI	I.Prior neurosurgical procedures<br>II.Number of metastases is more than 8<br>III.Meningeal carcinomatosis
Liang et al.—2022 [14]	NRG Oncology CC001 phase 3 trial patients who has available pretreatment MRI scans with known metastatic tumors	I.Incomplete sequences<br>II.Slice thickness of >5.5 mm<br>III.Severe artifacts in imaging<br>IV.Errors in the image standardization
Park et al.—2021 [39]	.Newly developed brain metastasis or without a brain metastasis with age and sex matching	.History of surgery or primary brain tumor<br>Gamma knife surgery or whole brain radiotherapy before MRI exam<br>III.Severe artifacts<br>IV.No follow-up MRI
Pennig et al.—2021 [40]	MR imaging scans at diagnosis of brain metastases<br>II.Distinct therapy after diagnosis of brain metastases<br>.The availability of all required MR imaging sequences	I.Presence of a second malignant tumor<br>II.Large intracranial extralesional bleeding<br>I.Acute ischemic stroke restricting delineation of brain metastases<br>IV.Severe artifacts<br>V.Insufficient contrast agent application
Pflüger et al.—2022 [41]	I.Adult patients with brain metastases who underwent standardized MRI examination for radiation treatment planning	Not reported

Rudie et al.—2021 [42]	I.Patients undergoing stereotactic radiosurgery planning	.Patients without definitive enhancing intracranial metastases<br>.Patients presented with only dura-based or leptomeningeal metastases<br>III.Scans that had missing sequences or yielded corrupted data
Xue et al.—2020 [43]	.Histopathological results of the primary tumor lesion available<br>II.Patients with only metastatic lesions in brain<br>III.Age over 18 years old<br>IV.3D T1 MPRAGE sequence is available	I.Skull and meningeal metastases<br>I.Poor imaging quality of 3D T1 MPRAGE<br>III.Missing data
Yin et al.—2022—Metastasis patients [44]	.Patients with extracranial primary tumor(s) confirmed by pathology<br>I.Patients with newly developed brain metastases<br>II.Patients underwent 3D-enhanced brain MRI and at least one follow-up MRI	.Patients with primary intracranial tumor(s)<br>II.Patients with meningeal metastasis<br>III.Patients undergone brain surgery<br>IV.Severe artifacts
Yin et al.—2022—Healthy controls [44]	.Patients with extracranial primary tumor(s) confirmed by pathology<br>II.Patients underwent 3D-enhanced brain MRI and showed no evidence of metastasis	.Patients with primary intracranial tumor(s)<br>II.Patients with meningeal metastasis<br>III.Patients undergone brain surgery<br>IV.Severe artifacts
Yoo et al.—2022 [45]	I.Patients with brain metastases who were treated with radiotherapy	Not reported
Yoo et al.—2021 [46]	I.Patients with brain metastases who were treated with stereotactic radiosurgery with 3D CE T1WI	I.Uncontoured metastases<br>II.Non-standard orientation<br>III.Non-standard FOV<br>IV.Artifacts
Zhang et al.—2020 [47]	Patients known to have definite clinical and imaging diagnosis of brain metastases	I.Patients with lesions not definitively known to represent metastases
Zhou et al.—2020 [48]	I.Patients who had undergone treatment planning for stereotactic radiosurgery by a board-certified radiation oncologist	I.Patients with a history of primary brain cancers or previous surgeries

CE, contrast-enhanced; WI, weighted imaging; MRI, magnetic resonance imaging; FLAIR, fluid attenuated inversion recovery; VISIBLE, volume isotropic simultaneous interleaved bright-blood and black-blood examination; BB, black blood; MPRAGE, magnetization prepared rapid gradient echo; FOV, field-of-view.
